# Dietary practices and associated factors during pregnancy in northwestern Ethiopia

**DOI:** 10.1186/s12884-018-1822-1

**Published:** 2018-05-25

**Authors:** Amanuel Nana, Tona Zema

**Affiliations:** 1 Faculty of Chemical and Food Engineering, Bahir Dar Institute of Technology, Bahir Dar University, Bahir Dar, Ethiopia; 2 School of Nutrition, Food Science and Technology, Hawassa University, Hawassa, Ethiopia

## Abstract

**Background:**

Pregnancy is the most crucial nutritionally demanding period of every woman’s life. The high demand of nutrients to deposit energy in the form of new tissue, growth of existing maternal tissues such as breast and uterus and increased energy requirements for tissue synthesis makes pregnant women more vulnerable to malnutrition. Dietary practice is defined as an observable actions or behavior of dietary habit and can be classified as good dietary practices and poor dietary practices. The incidence of dietary inadequacies as a result of dietary habits and patterns in pregnancy is higher during pregnancy when compared to any other stage of the life cycle. Thus, this study aimed to assess dietary practices and associated factors during pregnancy in Bahir Dar town, Northwest Ethiopia.

**Methods:**

A community based cross sectional study was conducted from March 1 to April 1, 2016. A total of 616 pregnant women were participated in the study. All eligible pregnant women were identified through house-to-house visit with the help of health extension workers. Cluster sampling was used to select eligible pregnant women. The data were collected using interviewer administered questionnaire prepared in English and translated in to Amharic. Data were analyzed by using Statistical Package for Social Sciences (SPSS) version 20. Multivariate logistic regression analysis was employed to identify factors associated with dietary practices.

**Result:**

This study has shown that 39.3% of the study participants had good dietary practices and the rest 60.7% of pregnant women reported poor dietary practices. Concerning dietary knowledge, 61.4% of the study participants had good dietary knowledge while 38.6% had poor dietary knowledge. Husband income, ownership of radio, history of disease and dietary knowledge were shown to have significant association (*P* < 0.05) with dietary practices.

**Conclusion:**

Dietary practices of pregnant women in the study area was suboptimal. Husband income, ownership of radio, history of disease and dietary knowledge were independent predictors of women dietary practices. Awareness should be created among pregnant women by concerned bodies such as governmental and non-governmental organization through local mass media.

## Background

Pregnancy is the most crucial nutritionally demanding period of every woman’s life. The high demand of nutrients to deposit energy in the form of new tissue, growth of existing maternal tissues such as breast and uterus and increased energy requirements for tissue synthesis makes pregnant women more vulnerable to malnutrition [1].

In 2010, maternal and child undernutrition was responsible for 1.5 million mortalities in the world [2]. A serious problem of maternal under nutrition is evident in most countries in sub-Saharan Africa, South-central and Southeastern Asia [3]. Ethiopia is one of the countries with very high burden of maternal and child under nutrition. Based on 2014 Ethiopian democratic and health survey (EDHS), maternal mortality (number of deaths per 100,000 live born) was 25.1% [4]. In 2011, 27% of Ethiopian women were undernourished and it is 29.8% in Amhara region where current study was conducted. Moreover, 23% of Ethiopian women aged 15–49 are anemic and 1% having severe anemia [5]. Malnutrition during pregnancy yield both short and long-term effects on the health of an infant by programming the infant’s development [6] and associated with risk of non-communicable diseases such as obesity, type 2 diabetes, hypertension and cardiovascular disease in later life [7]. Malnourished mothers are more vulnerable to diseases, encounter more miscarriages and give birth to underweight children whose survival is at risk [8, 9].

Pregnant women must get all essential nutrients and gain sufficient weight, the two main modifiable risk factors influencing maternal and infant outcomes [10]. Appropriate nutrition and weight gain benefits pregnant women to meet the demands of her offspring, her own body needs, and to prepare her body for lactation [11]. Low weight gain during pregnancy is risk factors for the delivery of infants too small for gestational age leading to neonatal mortality and morbidity [12], failure to grow, slow cognitive development and chronic diseases in adulthood [13].

Dietary practice is defined as observable actions or behavior of dietary habit and can be classified as having good dietary practices and poor dietary practices. The incidence of dietary inadequacies as a result of dietary habits and patterns in pregnancy is higher during pregnancy when compared to any other stage of the life cycle. Different scholars discovered that many women in developing countries restrict their food intake during pregnancy for different reasons such as to have smaller infants because smaller infants will carry a lower risk of delivery complications [14], cultural reason and perceived severity of delivery complications because big babies make delivery difficult [15, 16]. Thus, low intake of essential nutrients such as protein, energy, vitamins C, Vitamin A and iron due to inappropriate nutrition practices together with environmental factors, socioeconomic factors and infections are common causes of maternal mortality, low birth weight and intrauterine growth retardation [17, 18].

Even though the existence of government health sector development programs, it was recognized that poor nutritional status of children and women continues to be a serious problem in Ethiopia. Limited studies explored factors influencing maternal dietary practices in different parts of the country. For instance, exposure to nutrition information, attitude towards specific dietary habits and nutrition knowledge [19], attending antenatal care [20], maternal education and income [19, 20] were explored as predictors of maternal dietary practices. In addition, age at first marriage, meal frequency [21], educational status [22], occupation of head of household, religion [23], maternal age and marital status [22, 23], were discovered as predictors of maternal nutritional status which in turn influence dietary practices of mothers. Identifying specific factors affecting maternal dietary practice is necessary and critical to design right intervention. Thus, this study was aimed to assess dietary practices and associated factors of pregnant women in Bahir Dar town, Northwest Ethiopia.

## Methods

### Study settings, design and period

A community based cross-sectional study was employed from March 1–April 1, 2016 in Bahir Dar town, northwest Ethiopia. The town is located 565 km Northwest of Addis Ababa, the capital of Ethiopia. Based on the 2015 population projection, the town has total population of 243,300 of which 8262 (3.4%) were pregnant women. In the town, there are 10 government health centers, one referral hospital and many private health facilities. The town has a latitude of 11°36′N 37°23′E, longitude of 11°36′N 37°23′E and an elevation of about 1800 m (5,906 ft) above sea level.

### Source population and study population

The source population was all pregnant women who resided in Bahir Dar town during the study period. The study population was all pregnant women who lived in randomly selected sub cities.

### Sample size and sampling techniques

The sample size was calculated using single population proportion formula. Sample size determination was based on previous research finding of prevalence of poor dietary practices (59.9%) during pregnancy in Gondar town, Northwest Ethiopia [19]. Using margin of error (0.05), critical value at 95% confidence level (Z_1-ᾳ/2_ = 1.96), design effect (DE = 1.5) and non-response rate (10%), the total sample size calculated based on statistical formula was 611. Even though calculated sample size was 611, the investigators included a total of 616 pregnant women lived in the three selected sub cities so as to not miss additional 5 women in the area. Among nine sub cities (clusters) in Bahir Dar town, three sub cities were selected by simple random sampling method. All eligible pregnant women were identified through house-to-house visit with the help of health extension workers. No pregnant women were excluded since they were apparently healthy (self-reported) and had no disability problem at the time of data collection. Thus, all pregnant women who lived in the three selected clusters were included.

### Data collection tools and procedures

The data were collected using structured interviewer administered questionnaire by six trained diploma nurses. Socio-demographic and socio-economic factors, obstetric factors, dietary knowledge, dietary practices and mid upper arm circumference (MUAC) were assessed.

Questionnaire for evaluating dietary practices was adopted from English published articles and contextualized to local situations. Ten questions were used to assess dietary practices. The score of dietary practices was obtained by summation of responses of each question. Each question was given one mark if the answers were correct, favorable or healthy for dietary practices. Zero score was given if the responses were wrong, unfavorable or unhealthy for dietary practices. Study participants were classified to have poor dietary practices if they correctly answer < 75% of practice questions and good dietary practices if they correctly answer ≥75% of questions [24, 25]. Dietary knowledge of pregnant women was assessed by constructing 10 questions which were assumed to assess dietary knowledge of pregnant women on the aspects of nutrition required during pregnancy. Similar questions were used by previous study in Guto Gida district of Oromia region and this study adopted some of them [26].

Nutrition status of pregnant women was determined by using non-stretchable measuring tape made for measuring MUAC of adults. MUAC value of the left arm was taken to nearest 0.1 cm with no clothing on the arm and done in triplicate for each respondent in order to ensure accuracy [27]. Respondents were classified as undernourished (MUAC< 23 cm) and normal (MUAC ≥23 cm) [28].

### Data quality assurance, processing and analysis

Questionnaire was first prepared in English, translated to local language (Amharic) and then re-translated back to English to keep consistency. Two days training was given to data collectors and supervisor. The questionnaire was pre-tested on 5% of the actual sample size. Data were monitored and checked for completeness on daily basis during data collection. The data were coded, checked for any missing and entered into Epi-Info version 7 and then exported to Statistical Package for Social Sciences (SPSS) version 20.0 for analysis. Continuous data were checked for normality using Kolmogrov–Smirnov test. Descriptive statistics such as frequency, percentage, mean and standard deviation were generated. Bivariate analysis was employed to identify factors associated with dietary practices. Variables with *p*-value less than 0.2 in bivariate analysis were entered into multivariate analysis as it controls confounders. The association between dietary practices and independent variables were determined using crude odd ratio (COR) and adjusted odds ratio (AOR) at 95% confidence interval and variables with probability value (p-value) less than 0.05 were considered as statistically significant predictor of dietary practices.

## Results

### Socio demographic and socioeconomic characteristics of the study participants

A total of 616 pregnant women participated in this study making the response rate 100%. The mean age of study participants was 28.79 ± 5.13. About 36.4% of study participants were between age ranges of 25–29. Majority (94.5%) were Amhara by ethnicity. Four hundred seventy-three (76.8%) were orthodox by religion. Ninety-eight percent of the participants were married. Concerning education status, (37.5%) were attained secondary school (9–12). Forty-two percent of study participants were house wives and only 12 % were involved in other works. More than half (54.2%) of the participants earn on average less than 1000 Ethiopian birr (ETB). Majority (93.8%) of participants reported that monetary decisions made by husband and wife together. Average family size in this study was 3.45 ± 1.25 individuals and majority (81.3%) has family size greater than 4. About 97.4, 83.1 and 86.0% of participants have mobile phone, radio and television respectively (Table 1).

**Table 1:** Socio-demographic and socio-economic characteristics of the pregnant women in Bahir Dar town, Northwest Ethiopia, 2016 (*n* = 616)

Variable		Frequency (N)	Percent (%)
Age	19–24	138	22.4
	25–29	224	36.4
	30–34	168	27.3
	> 35	86	14.0
Ethnicity	Amhara	582	94.5
	Others^a^	34	5.5
Religion	Orthodox	473	81.3
	Muslim	115	18.7
	Protestant, Catholic and Adventists	28	4.5
Education status	Unable to read and write	34	5.5
	Read and write	62	10.1
	Grade 1–8	98	15.9
	Grade 9–12	231	37.5
	Higher institution	191	31.0
Respondent occupation	House wife	260	42.2
	Employed	135	21.9
	Merchant	146	23.7
	Other^b^	75	12.2
Respondent monthly income (in Ethiopian Birr)	< 1000	334	54.2
	1000–2000	137	22.2
	> 2000	145	23.5
Marital status	Married	604	98.1
	Single/Divorced/widowed	12	1.9
Head of household	Husband	603	97.9
	Others^c^	13	2.1
Husband occupation	Employed	235	38.1
	Merchant	253	41.1
	Daily laborer	59	9.6
	Others^d^	69	11.2
Husband income (ETB)	< 1000	162	26.3
	1000–2000	339	55
	> 2000	115	18.7
Monetary decision maker	Husband and wife together	578	93.8
	Others^e^	38	6.2
Family size	Less than 4	501	81.3
	More than 4	115	18.7
Mobile	Yes	600	97.4
Radio	Yes	512	83.1
TV	Yes	530	86.0
Laptop	Yes	100	16.2

^a^Tigre, Oromo, Awi; ^b^daily labor/private; ^c^wife, grandfather, grandmother; ^d^farmer, private; ^e^husband alone or wife alone

### Obstetric and pregnancy related characteristics of the study participants

Concerning obstetric and pregnancy related characteristics, more than half (53.7%) of women were at third trimester followed by 40.1% of women who were at second trimester. With regard to women’s previous pregnancies, about 549 (89.1%) of women had 0–2 previous pregnancies. Concerning number of times, a women gave birth to child, 52.9% of women had less than 2 parities. Sixty-four percent of women had more than 5-years pregnancy interval whereas only 2.4% had less than 2-years pregnancy interval. As far as experiencing illness is concerned during pregnancy, 36.5% of women experienced certain illness a month prior to the survey (Table 2).

**Table 2:** Obstetric and related characteristics of the pregnant women in Bahir Dar town, Northwest Ethiopia, 2016 (n = 616)

Variables	Frequency (N)	Percent (%)
Trimester		
First	38	6.2
Second	247	40.1
Third	333	53.7
Previous pregnancy		
0–2	549	89.1
3–5	67	10.9
Parity (*n* = 427)		
< 2	226	52.9
2–5	153	35.8
> 5	48	11.3
Pregnancy interval (n = 427)		
< 2 years	10	2.4
3–5 years	143	33.5
> 5 years	274	64.1
History of illness		
Yes	225	36.5
No	391	63.5

### Dietary knowledge of study participants

In this study, 378(61.4%) of the study participants had good dietary knowledge and 238(38.6%) had poor dietary knowledge. Specifically, except for the knowledge about iodine source foods (48.7%), vitamin A source foods (64.1%) and iron source foods (60.4%), all other knowledge variables scored≥75% indicating good dietary knowledge (Table 3).

**Table 3:** Dietary knowledge of the pregnant women in Bahir Dar town, Northwest Ethiopia, 2016(n = 616)

Questions (statements)/variables	Yes		No	
	#	%	#	(%)
Food is important for growth and development of fetus.	610	99	6	1
Food is important for providing heat, energy and for the normal functioning of women’s body.	605	98.2	11	1.8
Food is important for fighting infection or disease.	605	98.2	11	1.8
Knowledge about balanced diet	510	82.8	106	17.2
Inadequate diet can cause miscarriage and still birth.	422	68.5	194	31.5
Knowledge about carbohydrate source foods	463	75.2	153	24.8
Knowledge about protein source foods	487	79.1	129	20.9
Knowledge about iron source foods	372	60.4	244	39.6
Knowledge about vitamin A source foods	395	64.1	221	35.9
Knowledge about Iodine source foods	300	48.7	316	51.3
Knowledge status	Good (score ≥ 75%) 378			61.4%
	Poor (score < 75%) 238			38.6%

Source: [26]

### Dietary practices of the study participants

Dietary practices of study participants were indicated in Table 4. The result indicated that 203(33%) study participants avoid certain foods, of which 74.4% avoids food due to religious reason. About 61.7% skip their usual meal and the most commonly skipped meal was breakfast. From 436 (70.8%) who took an additional meal; 210(48.17%) and 217(49.77%) took one and two additional meals respectively. Concerning consumption of macro and micro nutrient source foods 41.7, 38.8, and 77.4%, were consuming carbohydrate, protein and vitamin rich foods like fresh vegetables respectively. About three fourth (75.3%) of the study participants had practiced checking weight in current pregnancy. Generally, majority (60.7%) of the study participants had poor dietary practices and the remaining 39.3% of the study participants had good dietary practices (Table 4).

**Table 4:** Dietary practices of the pregnant women in Bahir Dar town, Northwestern Ethiopia, 2016(n = 616)

Questions/variables		Number (n = 616)	Percentage (%)
Avoid any food or diet in the current pregnancy	Yes	203	33.0
	No	413	67.0
Reasons of avoiding	Religion	151	74.38
	Culture	2	1.0
	To avoid big baby	17	8.37
	Labor difficulty	6	2.95
	Others (dislike, discomfort)	27	13.30
Dietary regimen during current pregnancy	Yes	414	67.2
	No	202	32.8
Skipping meal during current pregnancy	Yes	380	61.7
	No	236	38.3
Type of meal skipped	Breakfast	266	70
	Lunch	6	1.6
	Snack	93	24.5
	Dinner	15	3.9
Taking additional meal	Yes	436	70.8
	No	180	29.2
Number of additional meals	1.One	210	48.17
	2. Two	217	49.77
	3. Three and more	9	2.06
Took iron supplement	Yes	400	64.9
	No	216	35.1
Habits of eating snacks between meals	Yes	494	80.2
	No	122	19.8
Consumption/eating CHO rich foods daily	Yes	257	41.7
	No	359	58.3
Consumption/eating protein rich foods daily	Yes	293	38.8
	No	377	61.2
Consumption/eating fresh vegetables daily	Yes	477	77.4
	No	139	22.6
Consumption/eating fruits daily	Yes	429	69.6
	No	187	30.4
Following weight during pregnancy	Yes	464	75.3
	No	152	24.7
	Good (score ≥ 75%)	242	39.3
Overall dietary practices	Poor (score < 75%)	374	60.7

### Factors associated with dietary practices of pregnant women

In multivariate logistic regression analysis, husband income, ownership of radio, history of illness and dietary knowledge revealed significant association with dietary practices(*P* < 0.05).Those study participants whose husbands earn 1000–2000 ETB per month were 2.8 times more likely to have good dietary practice than those earning less than 1000 ETB (AOR = 2.84, 95%CI, 1.74, 4.62) where as women whose husbands earn more than 2000 ETB were 3.1 times more likely to have good dietary practice than those earning less than 1000 ETB (AOR = 3.12, 95% CI, 1.743, 5.586).

The study participants who own radio were 3.17 times more likely to have good dietary practices than their counterparts (AOR = 3.17, 95%CI, 1.76, 5.67). Relative to women experienced illness; women who didn’t experience illness were 1.7 times more likely to have good dietary practices (AOR = 1.73, 95% CI, 1.17, 2.56). The study participants who have good dietary knowledge were 3.86 times more likely to have good dietary practice than their counterparts (AOR = 3.86, 95% CI, 1.91, 4.29) (Table 5).

**Table 5:** Factors associated with dietary practices of the pregnant women in Bahir Dar town, Northwestern Ethiopia, 2016(n = 616)

Variables		Dietary practices		COR (95% CI)	AOR(95%CI)
		Good	Poor		
Husband income	< 1000	38	124	1	1
	1000–2000	145	194	2.439(1.599,3.721)*	2.837(1.743,4.618)*
	> 2000	59	56	3.438(2.053,5.757)*	3.120(1.743,5.586)*
Owning radio	Yes	223	289	3.452(2.038,5.848)*	3.168(1.761,5.699)*
	No	19	85	1	1
History of illness	Yes	72	153	1	1
	No	170	221	1.635(1.159,2.306)*	1.734(1.173,2.564)*
Dietary knowledge	Good	187	191	3.257(1.214,4.441)*	3.864(1.915,4.285)*
	Poor	55	183	1	1

*Significant at p < 0.05

## Discussion

Maternal dietary practices during pregnancy play an important role in determining the long-term health and nutritional status of both the mother and her growing fetus. Poor dietary practices during pregnancy may result in increased rates of stillbirths, premature birth, low birth weight, maternal and prenatal death. In this study only 39.3% of the study participants had good dietary practices. This figure is nearly similar with study in Gondar town, northwest Ethiopia [19], but slightly higher than study in Guto Gida district, western Ethiopia [16]. The differences in dietary practices among studies, particularly modest improvement in dietary practices may be due to Ethiopian government is currently promoting nutrition related interventions through health extension program, health facility nutrition services, community-based women development army program and active involvement of pregnant women in focused antenatal care as well as in one-five network meeting at community level.

Even though health sectors developing different health and nutrition programs, this result have shown that poor dietary practices during pregnancy is still problem in Ethiopia. In current study, maternal nutrition regarding specific dietary practices such as skipping usual diets, avoiding certain food items and consumption of fresh fruits and vegetables were sub optimal. Avoiding food in pregnancy repeatedly reported by pregnant women. In this study more than one third of the study participants reported to avoid food during pregnancy. This finding is in line with other study in Oromia region, western Ethiopia [16]. On the contrary, this study indicated lower prevalence of skipping meal, avoiding foods, taking no additional meals during pregnancy and consumption of fruits and vegetables compared to study in Wondo Genet district, southern Ethiopia [15]. The discrepancy might be attributable to differences in sample size, geographical location, socio-economic characteristics and cultural differences. It is also repeatedly reported that dietary practices can be influenced by culture, socioeconomic and environmental determinants [18, 29, 30]. Low prevalence of avoiding food due to cultural reason reported in this study was consistent with studies in Wondo Genet district, southern Ethiopia [15]; but it is in contrary with previous studies in Guto Gida district, Gondar town and Shashemene district [16, 19, 20]. This may also be attributed to differences in sample size, geographical location and socio-cultural differences. This may be because of what is taboo (culture) in one society is not taboo in other society particularly when the society is very diverse.

In this study, husband’s income was significantly associated with dietary practices of pregnant women. This might be due to the fact that, the more husband earns, the more he invests on family nutrition and health which in turn attributes to good dietary practices of family in general and pregnant women in particular. Furthermore, earnings can influence availability of resources which in turn improve access to a diversified diet and thus improve dietary practices. This study is in line with other studies which also reported that income has positive significant association with women dietary practices [19, 31].

Present study also revealed that pregnant women having good dietary knowledge were more likely to have good dietary practices compared to women who do not have dietary knowledge. This was also documented by study conducted in Gondar town where nutrition knowledge shown significant association with women dietary practices [19]. One study in America also indicated that women dietary knowledge was predictor of good dietary practices [24]. Statistical significant association, but not strong association between dietary knowledge and dietary practice was also indicated by one study in Singapore [30]. This may have explained as, when a woman had good dietary knowledge, she become more concerned to good dietary practices and immediately can put it in to practice. On the other hand, good knowledge about basic nutrients and balanced diet usually resulting in positive dietary practices which are important determinants of optimum health from conception until death [32].

Current study has revealed that owning radio was significantly associated with women’s dietary practices. This might be attributable to owning radio and television would help individuals and families to broadcast important nutrition information such as about good or favorable dietary practices and its importance. Research evidence also suggested that women exposed to nutrition information were more likely to have good dietary practices than women haven’t exposed to nutrition information [16]. Present study also documented that women who experience illness were more likely to have poor dietary practices relative to women who didn’t experience illness. This might be due to synergistic relationship between diet and disease since infection can decrease appetite and dietary intake as explained in malnutrition-infection vicious cycle.

## Conclusion

In this study only 39.3% of the study participants had good dietary practice indicating that the overall dietary practice of the pregnant women in Bahir Dar town is suboptimal and the rest 60.7% of pregnant women reported poor dietary practices. Concerning dietary knowledge, 61.4% of the study participants had good dietary knowledge while 38.6% had poor dietary knowledge. Husband’s income, dietary knowledge, possessing radio and history of illness were significantly associated with dietary practices of pregnant women in Bahir Dar town, Northwest Ethiopia.

Therefore, increasing household income through providing income generation activities on the behalf of the government should be created. Improving knowledge on nutrition through nutrition education and integrating key nutrition messages in already existing strategy should be strengthened. Furthermore, means of access to radio and television should be created as they can play key role in improvement of dietary practices. Prevention and treatment of illness during pregnancy should also be strengthened. Finally, women of child bearing age should be considered and basic education should be strengthened on the behalf of the government.

### Limitation of the study

Lack of standardized questionnaire at national level and failure to assess food intake in terms of specific nutrients consumed.

## Abbreviations

AOR: Adjusted odd ratio
COR: Crude odd ratio
EDHS: Ethiopian demographic and health survey
ETB: Ethiopian birr
SPSS: Statistical package for social sciences
